# A community-led intervention to build neighbourhood identification predicts better wellbeing following prolonged COVID-19 lockdowns

**DOI:** 10.3389/fpsyg.2022.1030637

**Published:** 2022-12-09

**Authors:** Tegan Cruwys, Polly Fong, Olivia Evans, Joanne A. Rathbone

**Affiliations:** Research School of Psychology, The Australian National University, Canberra, ACT, Australia

**Keywords:** social identity, social cure, social interventions, public mental health, social cohesion

## Abstract

**Introduction:**

A growing body of research supports the importance of social cohesion for population wellbeing. However, the majority of this research has been correlational, and rarely have interventions been evaluated.

**Method:**

We conducted a two-timepoint study investigating the role of Neighbour Day, a grass-roots, community-led intervention that seeks to build social cohesion across the population. Among a sample of 843, 125 were Neighbour Day participants while the remainder were not.

**Results:**

We found that, compared to non-participants, Neighbour Day participants had significantly higher neighbourhood identification, experienced greater social cohesion, and had larger neighbourhood social networks. Between timepoints, the majority of the sample experienced prolonged lockdowns to prevent COVID-19 transmission, and so unsurprisingly, wellbeing declined and psychological distress increased. However, Neighbour Day participants were protected against these negative mental health effects of lockdown. These benefits of Neighbour Day participation were mediated *via* neighbourhood identification.

**Discussion:**

Overall, the findings speak to the promise of large-scale interventions to build social identity, particularly due to their capacity to build resilience and protect people’s wellbeing during times of collective change or crisis.

## Highlights

- Neighbour Day is a social cohesion intervention with the strengths of being cost-effective, scalable, and readily tailored to diverse populations.
- We evaluated Neighbour Day in a unique sample of 843 people pre- and post- a prolonged COVID lockdown.
- Lockdown led to increased psychological distress and reduced wellbeing.
- Neighbour Day participation was associated with greater social cohesion, size of neighbourhood social networks, and neighbourhood identification.
- To the extent that Neighbour Day participation predicted greater neighbourhood identification, it also protected mental health.
- We argue for an increased emphasis on social identity theorising and measures in social cohesion interventions.

## Introduction

The importance of investing in social cohesion has become starkly apparent in the context of the COVID-19 pandemic. Each community’s capacity (or lack thereof) to cooperatively organise to manage this unanticipated threat rapidly translated into a steep gradient in the effectiveness of their COVID response. For example, regions of England which had greater social cohesion prior to the pandemic were more likely to show solidarity during COVID restrictions (Lalot et al., 2021). In Australia, there is evidence that social cohesion protected mental health in the context of lockdowns (O’Donnell et al., 2022) and was associated with greater vaccination intentions (Cárdenas et al., 2022). Despite the demonstrated importance of social cohesion, two major challenges remain for researchers and health promoters: (1) how to conceptualise social cohesion, and (2) how to build social cohesion interventions that are not just effective but also cost-effective, community-led, and scalable. This article tackles each of these challenges by applying a social identity approach to the evaluation of a large-scale social cohesion intervention.

### The challenge of conceptualising social cohesion

The first challenge is theoretical: how can we best conceptualise and measure the “active ingredient” that enables a socially cohesive community? Social cohesion is a broad umbrella term that encompasses a variety of overlapping concepts. For example, social network size, mutual trust, and social participation have received attention in previous work (Sampson, 2003; Cramm et al., 2013; Kingsbury et al., 2019; Kress et al., 2020; for a review of the literature see Schiefer and Van der Noll, 2017). Previous studies that assess social cohesion specifically in the context of threats such as natural disasters and pandemics have used a wide variety of measures including sense of community, neighbourhood attraction, and contact with neighbours (for a review, see Jewett et al., 2021). However, because it is such a broad construct, there is little consensus on what social cohesion means precisely and how this can be harness through interventions to improve health outcomes. However, we posit that *neighbourhood identification* holds promise as a key construct that can progress thinking in this regard. Neighbourhood identification is a more narrow and theoretically precise concept than social cohesion. It refers to the degree to which a person derives a sense of subjective self-definition and affiliation from their local place-based community (Fong et al., 2019b). Neighbourhood identity, while overlapping with concepts in the literature such as sense of community, place identity or place attachment (Lewicka, 2011), is distinct in its grounding in the *social identity approach*—an influential theoretical framework for understanding social relationships more generally.

In the past decade a growing body of evidence has emerged for a strong and robust link between social identities (those aspects of our self-definition we derive from membership in social groups) and mental health (Jetten et al., 2017; Haslam et al., 2018). This relationship has been scrutinised and found to be attributable specifically to social identities, rather than other kinds of social variables. For example, in a sample of disadvantaged people who joined new recreational activity groups (thereby gaining a new group membership), depression scores declined 20% in 3 months (Cruwys et al., 2014). However, not all attendees derived this benefit, and indeed, frequency of attendance at the groups alone did not explain unique variance in depression. Instead, it was only those people who felt a strong sense of social identification with their new group that experienced these mental health benefits. Thus, is it only when groups are psychologically meaningful and important to us (i.e., we identify with our groups) that they are able to support our mental health. Sani et al. (2012) similarly found that, in a military sample, frequency of contact with one’s social groups did not predict depression or stress, but social identification did. That is, those who identified more strongly with their social groups experienced lower levels of depression and stress.

Furthermore, several studies have compared the predictive power of different forms of social relationships head-to-head, with a particular focus on comparing *group* memberships with *individual* relationships. For example, Haslam et al. (2014) utilised ten measures of social relationships that were available in the English Longitudinal Study of Ageing (Banks et al., 2021). A principal components analysis found that these measures loaded onto two factors: social group ties (including group memberships, community participation, etc.) and interpersonal ties (including quality of relationships with one’s spouse, number of friends, etc.). When both factors were included in a model to predict decline in cognitive health in older people, only group ties were a significant predictor. Participants with more group ties experienced significantly less decline in their cognitive health relative to those with fewer group ties. In a similar vein, a study of high school students found that the number of group memberships that students had uniquely predicted their self-esteem, with more group memberships associated with higher self-esteem (Jetten et al., 2015). In contrast, students’ self-esteem was not associated with the number of interpersonal friendships they had.

What might explain this unique role of social identification, relative to other social constructs? Social identity encapsulates not just the experientially pleasant aspects of social interaction, but instead is literally the psychological representation of one’s membership in a social group (Turner et al., 1987). Tajfel (1978) defined it as that “part of an individual’s self-concept which derives from his membership of a social group (or groups), together with the value and emotional significance attached to this.” (p. 63). It is this *self-definition* element of social identity that distinguishes it from other candidate constructs including social cohesion and interpersonal ties. It is also the case that many of the aspects of social connection so often touted as important – like trust, support and belonging – actually flow from social identities. Indeed, rigorous experimental studies have found that these and other psychological resources are provided by social identification (Greenaway et al., 2015, 2016; Junker et al., 2019; Kyprianides et al., 2019). Importantly, the value of social identities for mental health has been borne out in intervention studies, with a recent meta-analysis finding that a broad spectrum of health outcomes are positively affected by social identity interventions (Steffens et al., 2021), including in the context of COVID lockdowns (Cruwys et al., 2021; Bowe et al., 2022).

Turning to neighbourhood identification in particular, previous research has found that people who more strongly identify with their neighbourhood have better mental health (Fong et al., 2019a; Bowe et al., 2020). Promising evidence suggests that these benefits are present among socioeconomically disadvantaged neighbourhoods (Mcnamara et al., 2013; Stevenson et al., 2014; Evans and Rubin, 2021), and in the context of both gentrification and de-gentrification (Fong et al., 2019b). Recent evidence suggests that neighbourhood identification can be modified with light-touch interventions (Cruwys et al., 2022a). In sum then, the weight of the evidence suggests that neighbourhood identification is a promising candidate variable for research seeking to advance social cohesion interventions.

### The challenge of developing effective social cohesion interventions

A second challenge faced by those who seek to improve social cohesion is practical: the need to develop and evaluate interventions that are not just effective but also (1) cost-effective, (2) co-designed, that is, representing and respecting the needs and agency of the target population at all stages of the intervention, and (3) accessible, reaching populations who are in greatest need at their times of greatest need (Glasgow et al., 2003; Durlak and DuPre, 2008; Proctor et al., 2009; Harden et al., 2018). While these features are important for all interventions, the latter two are particularly essential for social cohesion interventions, which are unlikely to achieve their goals without buy-in from the communities they seek to benefit. To tackle all these challenges simultaneously is a tall order. For instance, reach and co-design may sometimes work in competition, because interventions that have the greatest reach (e.g., media campaigns) are often a “one size fits all” model. The ideal intervention is one that is designed by and for each of the communities we might wish to reach with an intervention, while still achieving cost-effectiveness and scalability.

*Neighbour Day* is a model that can address both the theoretical and practical concerns reviewed above. Now in its 20th year in Australia, Neighbour Day is a campaign that advocates for and empowers people to organise bespoke activities in their local communities that build social cohesion (e.g., having a neighbourhood barbeque/picnic, home visits to elderly neighbours, setting up a WhatsApp group for their street). Neighbour Day is not organised centrally by any one organisation or group – instead, it is a grass-roots initiative that involves thousands of unique events being conceived and led by “hosts” (i.e., activity organisers) within each target community. This means that every event is different in its content and tailored to the specific community that it seeks to engage. Relationships Australia, the non-profit organisation that is the custodian of Neighbour Day, promotes the event and makes resources available to both individuals and other community organisations to facilitate Neighbour Day activities. Neighbour Day is also a scalable model of intervention, with Relationships Australia estimating that almost 300,000 people were involved in a Neighbour Day activity in 2019. Its community-led nature means that this number could easily be increased further with a relatively modest investment.

One previous quantitative evaluation of Neighbour Day has been published (Fong et al., 2021), which found that Neighbour Day hosts experienced increased neighbourhood identification, improved social cohesion, reduced loneliness, and these benefits were sustained and predicted wellbeing at 6 month follow-up. However, no available evidence has (a) examined Neighbour Day relative to a control group, (b) looked at whether these benefits generalise to attendees as well as hosts, and (c) included a baseline measure of wellbeing to ensure these benefits represented a change over time. As well as addressing these limitations, the current study was also an opportunity to evaluate whether Neighbour Day is a model of intervention that remained relevant in the context of the pandemic and associated restrictions (both formal and self-imposed) on face-to-face social activities.

### The current study

Here, we utilised a unique dataset to evaluate the psychological benefits of Neighbour Day. We surveyed participants and non-participants in Neighbour Day in June and November of 2021 – pre and post a period of highly restrictive and disruptive lockdowns in Australia in response to the COVID delta outbreak. While much of the world remained heavily impacted by COVID in the early months of 2021 (which was prior to widespread vaccine availability), Australia had largely suppressed community transmission during this period and, notwithstanding closed international borders, community life was able to be conducted in relative normality (including Neighbour Day 2021, which was held in late March). However, with the emergence of the COVID delta strain, Australian states embarked on a series of “snap” lockdowns (i.e., without warning) from late June 2021 in an attempt to suppress the outbreak. Many of these lockdowns became prolonged: in the case of the cities of Sydney, Melbourne, and Canberra, where approximately 41% of the Australian population reside, these lockdowns lasted over 10 weeks. Our survey was thus able to not only assess Neighbour Day’s effects relative to a control group, but to do so in the context of a significant threat to social connectedness that affected the whole population to varying degrees.

Building on the previous research, we made three hypotheses:

**H1:** Neighbour Day participation will be associated with social benefits, as indexed by (a) greater neighbourhood identification, (b) greater social cohesion, and (c) greater interpersonal ties to neighbours at T1. Sensitivity analyses will also evaluate whether these benefits were present after controlling for covariates, whether they were moderated by neighbourhood socioeconomic disadvantage, and whether host vs. attendee status conferred benefit to differing degrees.

**H2:** Mental health will decline in the sample overall during a period of prolonged COVID lockdowns (from T1 to T2).

**H3:** Neighbour Day participants will be buffered against the effect of COVID lockdowns on mental health (T2) to the degree that Neighbour Day facilitated a strong sense of neighbourhood identification at T1. A sensitivity analysis was also conducted to evaluate whether neighbourhood identification was unique in its mediating role of these mental health benefits, or whether social cohesion or interpersonal ties were similarly protective.

## Materials and methods

### Participants

The final sample included 843 people at Time 1 (T1), of whom 125 were Neighbour Day participants (14.8%). A total of 484 participants were retained at 6 months later at Time 2 (T2) (57.4%); 76 (16.1%) of these were Neighbour Day participants. Our analyses utilised all available data for each research question.

Descriptive characteristics for the sample are provided in Table 1. Participants were sampled from 564 different neighbourhoods around Australia (Neighbour Day participants from 96 different neighbourhoods), spanning all state and territories and all deciles of neighbourhood socioeconomic status. Neighbour Day participants were younger, and more likely to be employed and partnered, than non-participants. Other demographics did not differ between groups. For this reason, demographics were included as covariates in the tests of the hypotheses concerning the effect of Neighbour Day (H1 and H3).

**TABLE 1:** Socio-demographic summary (T1; *N* = 843).

	Neighbour Day participants	Non-participants
	
	*N* (%)	
	125 (14.8%)	718 (85.2%)
**Gender**		
Woman	99 (79.2%)	512 (71.3%)
Man	25 (20.0%)	196 (27.3%)
Non-binary, other, or preferred not to say	1 (0.8%)	9 (1.2%)
Relationship status*		
Married/*de facto*	95 (76.0%)	440 (61.3%)
Single/never married/divorced/widowed	29 (23.2%)	276 (38.4%)
Employment status*		
Employed full-time or part-time	87 (69.6%)	357 (49.7%)
Not working/retired/disability/student/carer	38 (30.4%)	361 (50.3%)
**Education**		
Less than year 12	4 (3.2%)	51 (7.1%)
Year 12 cert	3 (2.4%)	38 (5.3%)
Certificate/diploma	23 (18.4%)	151 (21.0%)
Some university	21 (16.8%)	93 (13.0%)
Bachelor	54 (43.2%)	247 (34.4%)
Post-graduate	20 (16.0%)	137 (19.1%)

	***M* (SD)**	

Age*	47.56 (14.00)	53.07 (14.72)
Neighbourhood socioeconomic status	6.78 (2.78)	6.36 (2.81)

Gender was coded as men = −1, women = 1, non-binary, other or preferred not to say = 0 for the purposes of analyses. While we acknowledge that this has limitations and does not adequately capture the experience of non-binary people, it allowed these participants to be retained in the analyses while providing a statistical contrast between men and women (see also American Psychological Association[APA], 2022).

*Differed significantly between groups in a Chi-square test (categorical variables) or *t*-test (continuous variables).

### Design and procedure

Recruitment at T1 was conducted *via* advertising through Facebook, posts on Neighbour Day social media, and email invitation to previous Neighbour Day participants. In addition, respondents who were Neighbour Day participants were encouraged to refer their networks to the survey. All Australian residents aged 18+ years were eligible to participate. Respondents of the survey at T1 who were interested in participating in the T2 survey were asked to provide their email address. Participation in both surveys was voluntary. To ensure sufficient power, our target sample size was at least 400 people, with a minimum of 100 people who had participated in Neighbour Day. However, given the community-based nature of the research, no formal *a priori* power analysis was conducted.

**FIGURE 1:**
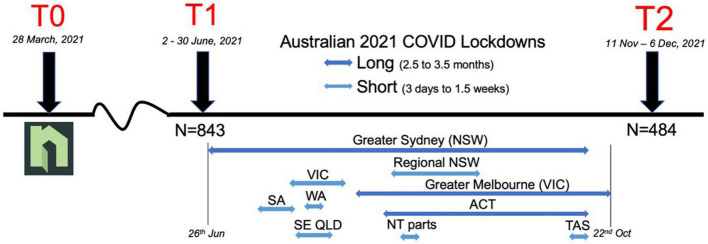
Timeline of data collection and COVID lockdown. Neighbour Day occurred at T0, survey data were collected at T1 and T2.

Between data collection timepoints in 2021, all of Australia’s states and territories were impacted by community transmission of COVID-19 (delta variant), see timeline in Figure 1. All states and territories of Australia imposed severe lockdown restrictions of various lengths lasting from 3 days and up to 3.5 months. Restrictions included the closure of most businesses and few legal reasons to leave one’s home (e.g., for essential work or medical care). By T2, almost all participants had experienced at least one lockdown, and 29.1% of respondents had been affected by a long lockdown (>2.5 months).

#### Survey incentives

Respondents went into a prize-draw to win one of five $100 (AUD) gift vouchers. Winners were randomly chosen from valid responders to the T1 survey. T1 responders were then invited to complete the T2 survey approximately 6 months later. The first 250 people to complete the T2 survey received a $20 shopping e-voucher, and all remaining T2 participants went into a prize-draw to win one of five Neighbour Day merchandise packages. Additionally, survey respondents at T1 who were Neighbour Day participants were offered incentives to refer the survey to others who completed the survey at T1. Specifically, respondents were offered $10 for 5 successful referrals, $20 for 10, or $50 for 20 additional survey respondents that they had referred.

#### Data preparation

Because of the considerable prize pool and snowball recruitment, a variety of strategies were implemented to identify and remove fraudulent responders. The survey software had inbuilt checks to counter “bot-like” responses (e.g., using captcha and identifying duplicate IP addresses). In addition, several attention check questions were included, which asked participants to respond to survey questions in particular ways (e.g., “Strongly Agree”) if they were paying attention. Finally, manual screening of the T1 data was undertaken to identify implausible responses based on demographics, postcode, email provided, survey timing, and IP addresses. The sample size of 843 participants represents the final sample of legitimate complete responses received at T1, with an additional 662 submitted surveys excluded.^1^

### Measures

Measures relevant to our hypotheses are detailed here, however the survey also included other items (primarily focused on descriptive information of Neighbour Day involvement) not relevant to this article. The full materials used in this research are available https://relationships.org.au/category/research/. Correlations are presented in Table 2.

**TABLE 2:** Correlations.

	1.	2.	3.	4.	5.	6.	7.	8.	9.	10.	11.	12.	13.
1. Age													
2. Gender	0.17*												
3. Education	−0.12*	–0.04											
4. Relationship status	0.00	–0.04	0.07*										
5. Employed	−0.39*	−0.09*	0.19*	0.16*									
6. Neighbourhood SES	−0.09*	−0.07*	0.15*	0.02	0.07*								
7. Neighbour Day participation (T0)	−0.13*	0.06	0.10	0.11*	0.14*	0.05							
8. Neighbourhood identification (T1)	0.18*	0.10*	0.00	0.16*	0.02	–0.01	0.20*						
9. Social cohesion (T1)	0.10*	0.02	0.02	0.16*	0.04	0.11*	0.09*	0.68*					
10. Neighbourhood interpersonal ties (T1)	0.18*	0.11*	–0.04	0.07	−0.07*	0.04	0.17*	0.38*	0.37*				
11. Wellbeing (T1)	0.18*	0.03	0.03	0.17*	0.03	0.04	0.11*	0.41*	0.35*	0.27*			
12. Wellbeing (T2)	0.17*	–0.01	0.03	0.15*	0.02	0.04	0.12*	0.37*	0.32*	0.24*	0.74*		
13. Psychological distress (T1)	−0.32*	–0.06	–0.04	−0.20*	–0.02	0.00	0.01	−0.32*	−0.30*	−0.20*	−0.65*	−0.55*	
14. Psychological distress (T2)	−0.34*	–0.03	–0.02	−0.16*	0.05	0.01	0.07	−0.31*	−0.26*	−0.14*	−0.62*	−0.62*	0.81*

Correlations are calculated based on available data; sample size ranged from 447 to 843.

*P < 0.05.

#### Neighbourhood identification

Participants’ sense of social identification with their neighbourhood was measured using the Four Item Social Identification scale (FISI; Doosje et al., 1995; Postmes et al., 2013). This scale has been widely used and validated for diverse populations and social groups. Items such as “I see myself as a resident of this neighbourhood” were rated on a scale from strongly disagree (1) to strongly agree (7), α_T1_ = 0.86.

#### Social cohesion

The degree to which participants perceived that their neighbourhood was cohesive was measured with a five-item scale from previous research (Sampson et al., 1997; Fong et al., 2021) that included both positively phrased items (e.g., “People in this neighbourhood can be trusted”), and negatively phrased items (e.g., “People in this neighbourhood do not get along with each other”), α_T1_ = 0.79.

#### Neighbourhood interpersonal ties

To assess the size of participants’ neighbourhood social network, participants were asked two questions (Cohen et al., 1997). First, participants selected as many options as applied to them from 12 categories in response to “My current social network (people you see or talk to at least once every 2 weeks) comprises of …” One option was “close neighbours.” For each of the categories selected, the following question asked “How many people do you have in each social network category?” where response options were limited to a numeric input. The number of close neighbours listed was used as the measure of neighbourhood interpersonal ties.

#### Psychological distress

The six-item Kessler-6 (K6; Kessler et al., 2003) was used to assess the severity of psychological symptoms indicative of probable mental illness. The K6 is a reliable and validated screener widely used in healthcare settings. The frequency of symptoms (e.g., nervousness and hopelessness) experienced in the past month were rated on a scale from none of the time (1) to all of the time (5), α_T1_ = 0.90; α_T2_ = 0.88.

#### Wellbeing

The five-item World Health Organization wellbeing scale (WHO-5; Bonsignore et al., 2001) was used to assess the degree to which participants had positive indicators of mental health. Participants rated the frequency in the past month of experiences such as “I have felt calm and relaxed” on a scale from at no time (0) to all of the time (5), α_T1and T2_ = 0.89. The overall scale was transformed to range from 0 to 100 in accordance with standard scoring.

#### Demographics

Participants provided their age, gender, level of education, relationship status, employment status, and postcode. Postcode was used to calculate decile of neighbourhood socioeconomic status (Australian Bureau of Statistics[ABS], 2018).

#### Neighbour Day experiences

To categorise respondents as Neighbour Day participants vs. non-participants, they were asked to indicate yes or no to the question “Did you participate in any events or activities related to Neighbour Day *this year* (2021)?” Those who answered yes were asked a follow up question: “if you did participate in 2021, were you a Neighbour Day …” with response option of (1) organiser, co-organiser, or host, (2) participant or attendee, or (3) other, please state. Five people selected “other,” but inspection of free responses suggested that they all contributed to hosting an event (e.g., “served sausages at neighbour day event”) and so were re-categorised as hosts.

## Results

### H1: Social benefits of Neighbour Day

Confirming H1a, a *t*-test found that Neighbour Day participation at T0 predicted a significantly greater sense of neighbourhood identification at T1 (*M* = 5.86; SD = 0.91), *t*(840) = 5.88, *d* = 0.57, *p* < 0.001 than among non-participants (*M* = 5.17; SD = 1.27). In a regression analysis controlling for the six covariates, support for H1a was replicated, with Neighbour Day participation significantly predicting neighbourhood identification and improving the model, β = 0.21, *R*^2^_change_ = 0.04, *F*_change_(1,740) = 33.38, *p* < 0.001. Among the covariates, older age (β = 0.23, *p* < 0.001) and being partnered (β = 0.12, *p* < 0.001) were associated with higher neighbourhood identification.

Confirming H1b, Neighbour Day participants (*M* = 5.15; SD = 1.07) reported significantly greater social cohesion in their neighbourhood, *t*(841) = 2.53, *d* = 0.25, *p* = 0.012 than non-participants (*M* = 4.88; SD = 1.12). In a regression analysis controlling for the six covariates, the effect of Neighbour Day participation on social cohesion marginally improved the model, β = 0.07, *R*^2^_change_ < 0.01, *F*_change_(1,740) = 3.26, *p* = 0.070. Among the covariates, older age (β = 0.14, *p* < 0.001) being partnered (β = 0.13, *p* < 0.001), and living in a higher socioeconomic status neighbourhood (β = 0.13, *p* < 0.001) were associated with greater social cohesion.

Confirming H1c, Neighbour Day participants reported having a larger number of neighbourhood interpersonal ties (*M* = 3.83; SD = 6.36), *t*(841) = 5.05, *d* = 0.49, *p* < 0.001 compared to non-participants (*M* = 1.92; SD = 3.29). In fact, non-participants were 30% more likely to report that they counted zero of their neighbours among their social network. In a regression analysis controlling for the six covariates, support for H1c was replicated, with Neighbour Day participation significantly predicting neighbourhood interpersonal ties and improving the model, β = 0.19, *R*^2^_change_ = 0.04, *F*_change_(1,740) = 28.75, *p* < 0.001. Among the covariates, older age (β = 0.18, *p* < 0.001) and being partnered (β = 0.07, *p* = 0.048) were associated with more neighbourhood interpersonal ties.

PROCESS (Model 1; Hayes, 2017) was used to conduct regression analyses to evaluate whether neighbourhood socioeconomic status moderated the effect of Neighbour Day participation on the three outcomes of neighbourhood identification, social cohesion, and neighbourhood interpersonal ties. Neighbour Day participation was specified as the independent variable and neighbourhood socioeconomic status as the moderator, with the remaining five covariates also included in analysis. Replicating the test of H1a, Neighbour Day participation significantly predicted neighbourhood identification, *B* = 0.62, SE = 0.31, *p* = 0.046. The interaction with neighbourhood socioeconomic status was not significant, *B* = 0.01, SE = 0.02, *p* = 0.914. The interaction was also not significant for the analyses predicting social cohesion (*B* = 0.03, SE = 0.04, *p* = 0.488) and interpersonal ties (*B* = 0.10, SE = 0.12, *p* = 0.429). This suggested no substantive differences in the benefits of Neighbour Day participation across neighbourhoods diverse in terms of their socioeconomic status.

Finally, *t*-tests were used to compare Neighbour Day hosts versus attendees to assess whether the social benefits derived (H1) were different for these two subgroups. No significant differences were found between these groups in neighbourhood identification, *t*(123) = 1.60, *p* = 0.113, social cohesion, *t*(123) = 0.94, *p* = 0.351, or interpersonal ties, *t*(123) = −0.96, *p* = 0.721.

### H2: Mental health costs of COVID lockdown

To assess H2, two paired samples *t*-tests were conducted. On average respondents experience a significant decline in their wellbeing from T1 to T2, *t*(467) = 3.91, *d* = 0.18, *p* < 0.001. Furthermore, on average respondents experienced a significant increase in psychological distress from T1 to T2, *t*(467) = −2.14, *d* = −0.10, *p* = 0.033. These effects were consistent with H2, that mental health would decline in the sample during prolonged COVID lockdowns.

### H3: Protective effect of Neighbour Day for mental health

To assess H3, two mediation analyses were specified in PROCESS (Model 4 with 5,000 bootstraps; Hayes, 2017). Neighbour Day participation (T0) was the independent variable, neighbourhood identification (T1) was the mediator, and psychological distress and wellbeing (T2) were the dependent variables. The corresponding measure of distress/wellbeing at T1 was included as a covariate in the relevant analysis, such that these analyses modelled the degree of change in the dependent variable, over and above its T1 level. Additionally, the six demographic covariates were included: age, gender, relationship status, employment status, education, and neighbourhood socioeconomic status.

**FIGURE 2:**
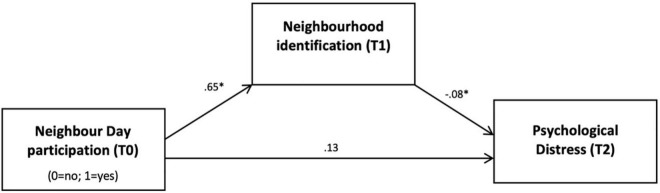
Neighbour Day participation protects against distress following COVID lockdown *via* neighbourhood identification. *N* = 440. Indirect effect was significant, –0.05 (–0.10, –0.01). Standardised beta coefficients are provided for each pathway in the model, *p < 0.05. Total effect was non-significant, β = 0.08, *p* = 0.334. Covariates included were psychological distress at T1, age, gender, relationship status, employment status, education, and neighbourhood socioeconomic status.

In the model predicting psychological distress (see Figure 2), Neighbour Day participation (T0) was significantly and positively related to neighbourhood identification at T1, β = 0.65, *p* < 0.001, and neighbourhood identification (T1), in turn, significantly and negatively predicted psychological distress at T2, β = −0.08, *p* = 0.009. Among the covariates, only T1 psychological distress was significant, β = 0.75, *p* < 0.001. Crucially for H3, the indirect effect was significant and negative, β = −0.05 (95% CI: −0.10, −0.01).

**FIGURE 3:**
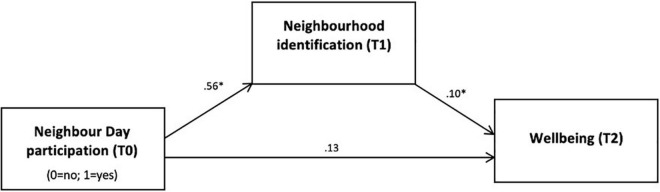
Neighbour Day participation protects wellbeing following COVID lockdown *via* neighbourhood identification. *N* = 440. Indirect effect was significant, 0.05 (0.01, 0.10). Total effect was significant, β = 0.18, *p* = 0.041. Fully mediated *via* neighbourhood identification. Standardised beta coefficients are provided for each pathway in the model, *p < 0.05. Covariates included were wellbeing at T1, age, gender, relationship status, employment status, education, and neighbourhood socioeconomic status.

**FIGURE 4:**
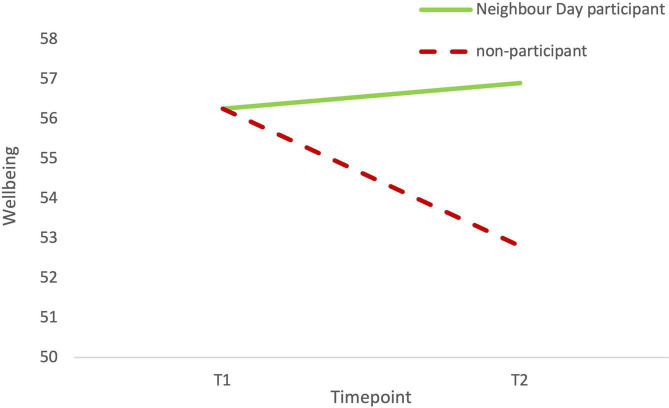
Neighbour Day participants were protected against a decline in wellbeing between T1 and T2 that was significant in the sample overall. *N* = 440. The figures reproduced the estimated marginal means from a regression analysis including the covariates of wellbeing at T1, age, gender, relationship status, employment status, education, and neighbourhood socioeconomic status.

In the model predicting wellbeing (see Figure 3), Neighbour Day participation (T0) was significantly and positively related to neighbourhood identification at T1, β = 0.56, *p* < 0.001, and neighbourhood identification (T1), in turn, significantly and positively predicted wellbeing at T2, β = 0.10, *p* = 0.007. None of the covariates significantly predicted T2 wellbeing except T1 wellbeing, β = 0.31, *p* < 0.001. The total effect of Neighbour Day participation on T2 wellbeing was significant, β = 0.18, *p* = 0.041. This effect was fully mediated *via* neighbourhood identification such that the indirect effect was significant and positive, β = 0.05 (95% CI: 0.01, 0.10). Thus, consistent with H3, the strong sense of neighbourhood identification at T1 that arose from Neighbour Day participation acted as a buffer against the threat of prolonged COVID lockdowns on mental health, compared to non-Neighbour Day participants. Figure 4 depicts the total effect from this analysis of Neighbour Day participation on wellbeing at T2.

Finally, as a sensitivity analysis, we replaced the mediator in these analyses with social cohesion or interpersonal ties. In each case, the indirect effect was non-significant for both wellbeing (β_social cohesion_ = 0.01, CI: −0.01, 0.03; β_interpersonal ties_ = 0.03, CI: −0.01, 0.06) and psychological distress (β_social cohesion_ = −0.01, CI: −0.03, 0.01; β_interpersonal ties_ = 0.01, CI: −0.02, 0.04). This suggested, consistent with our theorising, that the benefits of Neighbour Day participation for mental health were mediated *specifically* through their effects on social identity processes, rather than being more generalised.

## Discussion

This study evaluated the benefits of a bottom-up community initiative (Neighbour Day) to increase social cohesion through informal activities that bolster neighbourhood identification. We found that both hosts and attendees of Neighbour Day events had stronger neighbourhood identification, more social cohesion, and a greater number of interpersonal ties to neighbours, compared to the non-participating comparison group. These benefits were not moderated by neighbourhood socioeconomic status and were comparable in size for both hosts and attendees of Neighbour Day. Due to the COVID-delta outbreak in Australia and the highly restrictive lockdowns that ensued between T1 and T2 of measurement, we were able to assess the degree to which prior participation in Neighbour Day was protective against lockdown-related declines in mental health. While the sample overall did experience increased distress and reduced wellbeing at follow-up, Neighbour Day participation buffered against this. Consistent with our theorising, these benefits were fully mediated *via* neighbourhood identification, but not social cohesion or number of neighbourhood interpersonal ties.

This study went beyond previous work in several ways. First, while many interventions to bolster social cohesion have been attempted (e.g., Shen et al., 2017; Williams et al., 2020), few have been solidly informed by a theoretical framework and sought to target social identification as the key variable to achieve positive change. Neighbour Day is unique in doing so and, unlike other social identity interventions that have been evaluated, has great potential to reach whole communities and populations due to its grass-roots, scalable nature. One previous evaluation of Neighbour Day has been conducted (Fong et al., 2021), and its findings accord with those here. However, this previous analysis lacked a comparison group, focused solely on hosts rather than attendees, and did not collect a baseline measure of wellbeing in order to consider change. It also did not examine the benefit of Neighbour Day for populations “under pressure.” Whereas in the current study, we were able to demonstrate that communities affected by pandemic-related restrictions were protected from the widespread decline in mental health that occurred in the wake of such challenges. The present study therefore provides crucial evidence for establishing the promise of Neighbour Day as a model for social cohesion interventions because not only does participation bolster identification, enhance a sense of cohesion and increase neighbourhood ties, it can also protect wellbeing.

### Implications

From a practical standpoint, this study has several implications for those who seek to improve social cohesion. It provides an example of how a community-led, theoretically informed intervention can be effective in delivering both social and health benefits to diverse populations. The findings also suggest that it is possible for interventions to be simultaneously co-designed and accessible, while also being most likely cost-effective. A formal economic analysis is required, however, to evaluate Neighbour Day in terms of its scalability, cost-effectiveness, and accessibility. Finally, it speaks to the importance of investing in community ties “while the sun is shining,” and that this will likely pay off when communities fall upon unexpected hard times. The benefits of Neighbour Day were not just apparent for well-off folk in positive circumstances, but were also seen in more disadvantaged communities during a time of unanticipated stressors on communities.

This study also has theoretical implications for several audiences. For social cohesion researchers, we provide evidence that, consistent with our theorising, neighbourhood identification was a key driver of mental health outcomes. Neither our direct measure of social cohesion nor our measure of interpersonal ties to neighbours explained the benefits of Neighbour Day participation for mental health. We predicted this due to social identity’s unique focus on capturing a felt sense of self-definition informed by a place-based community. Future research should continue to explore this by ensuring that a measure of people’s subjective sense of connection to their place-based community (i.e., neighbourhood identification) is included in evaluations.

For social cure researchers, this study illustrates the importance of considering cost and scalability in the design of interventions. These may be major strengths of social cure interventions that have not been fully appreciated in prior work, and attention to this could help this new generation of health interventions to avoid some of the pitfalls faced by previous frameworks (e.g., by developing interventions that are resource-intensive and not scalable).

Finally, for mental health researchers and health promoters, a key implication of this research is that non-clinical interventions embedded in the social context of people’s lives can achieve substantial and sustained benefit in reducing psychological distress and improving wellbeing. Against a backdrop of clinician shortages and long wait times for clients to access mental health services, the value of community-based interventions for reducing population mental health burden should not be overlooked. Collective events, which include Neighbour Day but also other gatherings and rituals, can be a way for group members to *enact* their shared identity in ways that serve to both reinforce it and benefit their wellbeing (Khan et al., 2016; Cruwys et al., 2019; Morton and Power, 2022). It is also germane to consider the growing evidence base for how social identification can be actively shaped and supported through intervention. In previous social identity interventions (see Steffens et al., 2021), effective strategies have included shared group activities, common goals, an emphasis on similarities among group members (versus distinctiveness from other groups), and a focus on enabling the group to endure into the future.

### Strengths, limitations, and future directions

This study had several strengths including its large and diverse sample at two timepoints, the use of validated measures of the focal constructs, and the inclusion of a comparison group of people who had not participated in the intervention. However, an important weakness of the design is that the intervention took place prior to T1, and so from these data alone we cannot be confident that the differences between Neighbour Day participants and non-participants were an outcome of participation. For this reason, these findings are best understood in concert with the broader social cure literature, which includes experiments and clinical trials (Cruwys et al., 2022a,b). One gap that future research should prioritise is how to reach groups that are less likely to voluntarily engage with Neighbour Day. In this sample, older people who were single and not working were less likely to participate. This is concerning, as people from these demographics are known to be at an elevated risk for loneliness and are also likely to benefit most from interventions that increase their social connections (Berg-Weger and Morley, 2020; Morrish and Medina-Lara, 2021). Recent qualitative research provides some insights into the challenges of engaging this group in social interventions (Stuart et al., 2022), but more research is needed to establish how these barriers can be overcome.

## Conclusion

Across the world, health systems are struggling to respond to demand for mental health services in the wake of the COVID-19 pandemic. Innovative approaches are needed to support population mental health that are scalable, accessible, and readily tailored to the needs of specific groups. This study presented evidence that Neighbour Day – a campaign that supports local communities to connect with one another – is effective in improving social cohesion, neighbourhood identification, and building social networks in neighbourhoods. Neighbour Day participants were protected against a population-level decline in mental health that occurred in the context of the pandemic, and this was uniquely mediated *via* neighbourhood identification. We hope this evidence acts as an impetus for researchers and practitioners to prioritise developing and evaluating accessible interventions that increase social identity.

## Data availability statement

The raw data supporting the conclusions of this article will be made available by the authors, without undue reservation.

## Ethics statement

The studies involving human participants were reviewed and approved by the Human Research Ethics Committee at The Australian National University (2019/132). The patients/participants provided informed consent to participate in this study.

## Author contributions

TC led the development of the research question, led the grant that enabled the research, conducted the final analyses, and wrote the manuscript. PF led survey design, data collection and management, and conducted the initial analyses. All authors contributed to the conceptualisation of the research and provided constructive feedback on the manuscript.

## Footnotes

1 While this may seem like a large proportion, a few instances of easily identified fraud (because we offered a $50 gift e-voucher for 20 successful survey referrals) accounted for the majority of these cases – with identical free responses, postcodes, and IP addresses, completed in quick succession at unlikely times of the day. Because people were required to provide a referral code, these fraudulent responses were readily linked to the referrer and excluded. We also conducted exhaustive manual checking of the data to ensure retained responses were valid.

## References

[B1] American Psychological Association [APA] (2022). *APA style guide: Gender.* Washington, DC: American Psychological Association.

[B2] Australian Bureau of Statistics [ABS] (2018). *Census of population and housing: Socio-economic indexes for areas (SEIFA), Australia, 2016.* Canberra, ACT: Australian Bureau of Statistics.

[B3] Banks J. Batty G. D. Breedvelt J. J. F. Coughlin K. Crawford R. Marmot M. (2021). *English longitudinal study of ageing: Waves 0-9, 1998-2019 [data collection] SN: 5050*, 36th Edn. Colchester: UK Data Service. 10.5255/UKDA-SN-5050-23

[B4] Berg-Weger M. Morley J. E. (2020). Loneliness in old age: An unaddressed health problem. *J. Nutr. Health Aging* 24 243–245. 10.1007/s12603-020-1323-6 32115602 PMC7223173

[B5] Bonsignore M. Barkow K. Jessen F. Heun R. (2001). Validity of the five-item WHO Well-Being Index (WHO-5) in an elderly population. *Eur. Arch. Psychiatry Clin. Neurosci.* 251 27–31. 10.1007/bf03035123 11824831

[B6] Bowe M. Gray D. Stevenson C. McNamara N. Wakefield J. R. H. Kellezi B. (2020). A social cure in the community: A mixed-method exploration of the role of social identity in the experiences and well-being of community volunteers. *Eur. J. Soc. Psychol.* 50 1523–1539. 10.1002/ejsp.2706

[B7] Bowe M. Wakefield J. R. H. Kellezi B. Stevenson C. McNamara N. Jones B. A. (2022). The mental health benefits of community helping during crisis: Coordinated helping, community identification and sense of unity during the COVID-19 pandemic. *J. Community Appl. Soc. Psychol.* 32 521–535. 10.1002/casp.2520 34230792 PMC8250747

[B8] Cárdenas D. Donaldson J. L. Donnell J. O. Stevens M. Cruwys T. Zekulin M. (2022). *Social cohesion at the outset of COVID-19 predicts later vaccination intentions.* Canberra, ACT: Australian National University.

[B9] Cohen S. Doyle W. Skoner D. Rabin B. Gwaltney J. Jr. (1997). Social ties and susceptibility to the common cold. *JAMA* 277 1940–1944. 9200634

[B10] Cramm J. M. Van Dijk H. M. Nieboer A. P. (2013). The importance of neighborhood social cohesion and social capital for the well being of older adults in the community. *Gerontologist* 53 142–150. 10.1093/geront/gns052 22547088

[B11] Cruwys T. Haslam C. Rathbone J. A. Williams E. Haslam S. A. (2021). Groups 4 Health protects against unanticipated threats to mental health: Evaluating two interventions during COVID-19 lockdown among young people with a history of depression and loneliness. *J. Affect. Disord.* 295 316–322. 10.1016/j.jad.2021.08.029 34488085 PMC8413117

[B12] Cruwys T. Fong P. Evans O. Batterham P. Calear A. L. (2022a). Boosting neighbourhood identification to benefit wellbeing : Evidence from diverse community samples. *J. Environ. Psychol.* 81:101816. 10.1016/j.jenvp.2022.101816

[B13] Cruwys T. Haslam C. Rathbone J. A. Williams E. Haslam S. A. Walter Z. C. (2022b). Groups 4 Health versus cognitive-behavioural therapy for depression and loneliness in young people: Randomised phase 3 non-inferiority trial with 12-month follow-up. *Br. J. Psychiatry* 220 140–147. 10.1192/bjp.2021.128 35049477

[B14] Cruwys T. Haslam S. A. Dingle G. A. Jetten J. Hornsey M. J. Chong E. M. D. (2014). Feeling connected again: Interventions that increase social identification reduce depression symptoms in community and clinical settings. *J. Affect. Disord.* 159 139–146. 10.1016/j.jad.2014.02.019 24679402

[B15] Cruwys T. Saeri A. K. Radke H. R. M. Walter Z. C. Crimston D. Ferris L. J. (2019). Risk and protective factors for mental health at a youth mass gathering. *Eur. Child Adolesc. Psychiatry*. 28, 211–222. 10.1007/s00787-018-1163-7 29752533

[B16] Doosje B. Ellemers N. Spears R. (1995). Perceived intragroup variability as a function of group status and identification. *J. Exp. Soc. Psychol*. 31, 410–436.

[B17] Durlak J. A. DuPre E. P. (2008). Implementation matters: A review of research on the influence of implementation on program outcomes and the factors affecting implementation. *Am. J. Community Psychol.* 41 327–350. 10.1007/s10464-008-9165-0 18322790

[B18] Evans O. Rubin M. (2021). In a class on their own: Investigating the role of social integration in the association between social class and mental well-being. *Pers. Soc. Psychol. Bull.* 48 690–703. 10.1177/01461672211021190 34092129

[B19] Fong P. Cruwys T. Haslam C. Haslam S. A. (2019a). Neighbourhood identification buffers the effects of (de-)gentrification and personal socioeconomic position on mental health. *Health Place* 57 247–256. 10.1016/j.healthplace.2019.05.013 31128527

[B20] Fong P Cruwys T. Haslam C. Haslam S. A. (2019b). Neighbourhood identification and mental health: How social identification moderates the relationship between socioeconomic disadvantage and health. *J. Environ. Psychol.* 61 101–114. 10.1016/J.JENVP.2018.12.006

[B21] Fong P Cruwys T. Robinson S. L. Haslam S. A. Haslam C. (2021). Evidence that loneliness can be reduced by a whole-of-community intervention to increase neighbourhood identification. *Soc. Sci. Med.* 277:113909. 10.1016/j.socscimed.2021.113909 33866082

[B22] Glasgow R. E. Lichtenstein E. Marcus A. C. (2003). Why don’t we see more translation of health promotion research to practice ? rethinking the efficacy-to-effectiveness transition. *Am. J. Public Health* 93 1261–1267. 10.2105/ajph.93.8.1261 12893608 PMC1447950

[B23] Greenaway K. H. Cruwys T. Haslam S. A. Jetten J. (2016). Social identities promote well-being because they satisfy global psychological needs. *Eur. J. Soc. Psychol.* 46 294–307. 10.1002/ejsp.2169

[B24] Greenaway K. H. Haslam S. A. Cruwys T. Branscombe N. R. Ysseldyk R. Heldreth C. (2015). From “we” to “me”: Group identification enhances perceived personal control with consequences for health and well-being. *J. Pers. Soc. Psychol.* 109 53–74. 10.1037/pspi0000019 25938701

[B25] Harden S. M. Smith M. L. Ory M. G. Smith-Ray R. L. Estabrooks P. A. Glasgow R. E. (2018). RE-AIM in clinical, community, and corporate settings: Perspectives, strategies, and recommendations to enhance public health impact. *Front. Public Health* 6:71. 10.3389/fpubh.2018.00071 29623270 PMC5874302

[B26] Haslam C. Jetten J. Cruwys T. Dingle G. A. Haslam S. A. (2018). *The new psychology of health: Unlocking the social cure.* London: Routledge.

[B27] Haslam Catherine Cruwys T. Haslam S. A. (2014). “The we’s have it”: Evidence for the distinctive benefits of group engagement in enhancing cognitive health in aging. *Soc. Sci. Med.* 120 57–66. 10.1016/j.socscimed.2014.08.037 25222136

[B28] Hayes A. F. (2017). *Introduction to mediation, moderation, and conditional process analysis: A regression-based approach*, New York, NY: Guilford Press.

[B29] Jetten J. Haslam S. A. Cruwys T. Greenaway K. H. Haslam C. Steffens N. K. (2017). Advancing the social identity approach to health and well-being: Progressing the social cure research agenda. *Eur. J. Soc. Psychol.* 47 789–802. 10.1002/ejsp.2333

[B30] Jetten J. Branscombe N. R. Haslam S. A. Haslam C. Cruwys T. (2015). Having a lot of a good thing: Multiple important group memberships as a source of self-esteem. *PLoS One* 10:e0124609. 10.1371/journal.pone.0124609 26017554 PMC4446320

[B31] Jewett R. L. Mah S. M. Howell N. Larsen M. M. (2021). Social cohesion and community resilience during COVID-19 and pandemics: A rapid scoping review to inform the united nations research roadmap for COVID-19 recovery. *Int. J. Health Serv*. 51, 325–336. 10.1177/0020731421997092 33827308 PMC8204038

[B32] Junker N. M. van Dick R. Avanzi L. Häusser J. A. Mojzisch A. (2019). Exploring the mechanisms underlying the social identity–ill-health link: Longitudinal and experimental evidence. *Br. J. Soc. Psychol.* 58 991–1007. 10.1111/bjso.12308 30561049

[B33] Kessler R. C. Barker P. R. Colpe L. J. Epstein J. F. Gfroerer J. C. Hiripi E. (2003). Screening for serious mental illness in the general population. *Arch. Gen. Psychiatry* 60 184–189. 10.1001/archpsyc.60.2.184 12578436

[B34] Khan S. S. Hopkins N. Reicher S. Tewari S. Srinivasan N. Stevenson C. (2016). How collective participation impacts social identity: A Longitudinal study from India. *Polit. Psychol.* 37 309–325. 10.1111/pops.12260

[B35] Kingsbury M. Clayborne Z. Colman I. Kirkbride J. B. (2019). The protective effect of neighbourhood social cohesion on adolescent mental health following stressful life events. *Psychol. Med.* 50 1292–1299. 10.1017/S0033291719001235 31179962 PMC7322549

[B36] Kress S. Razum O. Zolitschka K. A. Breckenkamp J. Sauzet O. Sauzet O. (2020). Does social cohesion mediate neighbourhood effects on mental and physical health? Longitudinal analysis using German socio-economic panel data. *BMC Public Health* 20:1043. 10.1186/s12889-020-09149-8 32611338 PMC7328265

[B37] Kyprianides A. Easterbrook M. J. Brown R. (2019). Group identities benefit well-being by satisfying needs. *J. Exp. Soc. Psychol.* 84:103836. 10.1016/j.jesp.2019.103836

[B38] Lalot F. Abrams D. Broadwood J. Davies Hayon K. Platts-Dunn I. (2021). The social cohesion investment: Communities that invested in integration programmes are showing greater social cohesion in the midst of the COVID-19 pandemic. *J. Community Appl. Soc. Psychol.* 32 536–554. 10.1002/casp.2522 34230795 PMC8251431

[B39] Lewicka M. (2011). Place attachment: How far have we come in the last 40 years? *J. Environ. Psychol.* 31 207–230. 10.1016/j.jenvp.2010.10.001

[B40] Mcnamara N. Stevenson C. Muldoon O. T. (2013). Community identity as resource and context: A mixed method investigation of coping and collective action in a disadvantaged community. *Eur. J. Soc. Psychol.* 43 393–403. 10.1002/ejsp.1953

[B41] Morrish N. Medina-Lara A. (2021). Does unemployment lead to greater levels of loneliness? A systematic review. *Soc. Sci. Med.* 287:114339. 10.1016/j.socscimed.2021.114339 34455335 PMC8505794

[B42] Morton T. A. Power S. A. (2022). Coming together after standing apart: What predicts felt safety in the post-coronavirus crowd? *Soc. Sci. Med.* 293:114649. 10.1016/j.socscimed.2021.114649 34906827 PMC8665825

[B43] O’Donnell J. Cárdenas D. Orazani N. Evans A. Reynolds K. J. (2022). longitudinal effect of COVID-19 infections and lockdown on mental health and the protective effect of neighbourhood social relations. *Soc. Sci. Med.* 297:114821. 10.1016/j.socscimed.2022.114821 35219050 PMC8847081

[B44] Postmes T. Haslam S. A. Jans L. (2013). A single-item measure of social identification: Reliability, validity, and utility. *Br. J. Soc. Psychol*. 52, 597–617. 10.1111/bjso.12006 23121468

[B45] Proctor E. K. Landsverk J. Aarons G. Chambers D. Glisson C. Mittman B. (2009). Implementation research in mental health services: An emerging science with conceptual, methodological, and training challenges. *Adm. Policy Ment. Health* 36 24–34. 10.1007/s10488-008-0197-4 19104929 PMC3808121

[B46] Sampson R. J. (2003). The neighborhood context of well-being. *Perspect. Biol. Med.* 46 S53–S64. 10.1353/pbm.2003.0073 14563074

[B47] Sampson R. J. Raudenbush S. W. Earls F. (1997). Neighborhoods and violent crime: A multilevel study of collective efficacy. *Science* 277 918–925. 9252316 10.1126/science.277.5328.918

[B48] Sani F. Herrera M. Wakefield J. R. H. Boroch O. Gulyas C. (2012). Comparing social contact and group identification as predictors of mental health. *Br. J. Soc. Psychol.* 51 781–790. 10.1111/j.2044-8309.2012.02101.x 22550954

[B49] Schiefer D. Van der Noll J. (2017). The essentials of social cohesion: A literature review. *Soc. Indic. Res.* 132 579–603. 10.1007/s11205-016-1314-5

[B50] Shen C. Wan A. Kwok L. T. Pang S. Wang X. Stewart S. M. (2017). A community-based intervention program to enhance family communication and family well-being: The learning families project in Hong Kong. *Front. Public Health* 5:251. 10.3389/fpubh.2017.00257 29085815 PMC5649187

[B51] Steffens N. K. LaRue C. J. Haslam C. Walter Z. C. Cruwys T. Munt K. A. (2021). Social identification-building interventions to improve health: A systematic review and meta-analysis. *Health Psychol. Rev.* 15 85–112. 10.1080/17437199.2019.1669481 31530154

[B52] Stevenson C. Mcnamara N. Muldoon O. (2014). Stigmatised identity and service usage in disadvantaged communities: Residents’, community workers’ and service providers’ perspectives. *J. Community Appl. Soc. Psychol.* 24 453–466. 10.1002/casp

[B53] Stuart A. Stevenson C. Koschate M. Cohen J. Levine M. (2022). ‘Oh no, not a group!’ The factors that lonely or isolated people report as barriers to joining groups for health and well-being. *Br. J. Health Psychol.* 27 179–193. 10.1111/bjhp.12536 34028949

[B54] Tajfel H. (1978). *Differentiation between social groups: Studies in the social psychology of intergroup relations.* London: Academic Press.

[B55] Turner J. C. Hogg M. A. Oakes P. J. Reicher S. D. Wetherell M. S. (1987). *Rediscovering the social group: A self-categorization theory.* Oxford: Blackwell.

[B56] Williams A. J. Maguire K. Morrissey K. Taylor T. Wyatt K. (2020). Social cohesion, mental wellbeing and health-related quality of life among a cohort of social housing residents in Cornwall: A cross sectional study. *BMC Public Health* 20:985. 10.1186/s12889-020-09078-6 32571296 PMC7310403

